# Local generalised method of moments: an application to point process‐based rainfall models

**DOI:** 10.1002/env.2338

**Published:** 2015-03-22

**Authors:** Jo M. Kaczmarska, Valerie S. Isham, Paul Northrop

**Affiliations:** ^1^ Department of Statistical ScienceUniversity College LondonGower StreetLondonWC1E 6BTU.K.

**Keywords:** nonparametric regression, smoothing, statistical downscaling, stochastic processes, weather generator

## Abstract

Long series of simulated rainfall are required at point locations for a range of applications, including hydrological studies. Clustered point process‐based rainfall models have been used for generating such simulations for many decades. These models suffer from a major limitation, however: their stationarity. Although seasonality can be allowed by fitting separate models for each calendar month or season, the models are unsuitable in their basic form for climate impact studies. In this paper, we develop new methodology to address this limitation. We extend the current fitting approach by allowing the discrete covariate, calendar month, to be replaced or supplemented with continuous covariates that are more directly related to the incidence and nature of rainfall. The covariate‐dependent model parameters are estimated for each time interval using a kernel‐based nonparametric approach within a generalised method‐of‐moments framework. An empirical study demonstrates the new methodology using a time series of 5‐min rainfall data. The study considers both local mean and local linear approaches. While asymptotic results are included, the focus is on developing useable methodology for a complex model that can only be solved numerically. Issues including the choice of weighting matrix, estimation of parameter uncertainty and bandwidth and model selection are considered from this perspective. © 2015 The Authors. *Environmetrics* Published by John Wiley & Sons Ltd.

## 1 Introduction

Rainfall series are required at a range of spatial and temporal scales by hydrologists, telecommunications engineers and those involved in the modelling of climate impacts on agriculture and the environment. These series typically need to cover very long periods into the future, and climate change is increasingly a concern. Observed series reflect only the historical climate, are generally too short to meet requirements and may suffer from quality issues. There is thus a need for models from which realistic artificial rainfall series can be simulated.

Generalised circulation models (GCMs) are the main tools for predicting future climate impacts resulting from the increase of greenhouse gases in the atmosphere. They model large‐scale movements over the entire globe over tens to hundreds of years using a set of physical equations. GCMs cannot reliably model precipitation, however, primarily because the low resolution means that details of local topography (such as mountains and coastline), which are important for rainfall, are lost. Even with the use of embedded regional climate models (RCMs), which cover smaller areas in more detail, the accuracy of these physical models is limited because of outstanding deficiencies in understanding of cloud formation and precipitation processes.

GCMs and RCMs can, however, be used to produce high‐resolution rainfall projections using a technique known as ‘statistical downscaling’, whereby observed relationships between the large‐scale climate variables of the GCM (or RCM) and local climate are exploited. For example, one such approach relates daily rainfall occurrence and conditional wet‐day rainfall amounts to climate variables using generalised linear models (Chandler & Wheater, 2002). This is effectively an extension of the two‐state (wet/dry) first‐order Markov chain model of Katz (1977) or Stern & Coe (1984) and is appropriate when rainfall series are required at a daily timescale. However, it is not feasible at subdaily resolution, as the complicated dependency structure of rainfall would require an excessive number of parameters.

**Figure 1:**
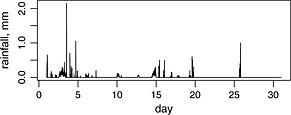
A sample of the Bochum series of 5‐min rainfall totals over January 1981

Data at subhourly resolution are required at point locations for various applications. For example, ‘rain fade’ is one of the main causes of outage of radio telecommunications networks, requiring mitigation strategies such as the incorporation of route diversity. In the hydrological field, data at fine‐scale are required for sewer systems and urban drainage design. Clustered point process‐based rainfall models have been used to address this requirement, particularly in the field of hydrology, since a seminal paper by Rodriguez‐Iturbe *et al.* (1987). There are two basic clustering mechanisms used and numerous versions of the models. All consist of a clustered point process of rain cell arrivals, together with a set of random variables that determine the durations and intensity profiles of the rain cells. This model structure is appealing as it reflects important aspects of the physical process: the fact that rainfall totals over short intervals are very often exactly zero (as can be seen in Figure 1), and the clustering exhibited by rainfall in both space and time (Austin & Houze, 1972). Because the underlying process runs in continuous time, another advantage of this type of model is that simulations can be generated that aggregate to different timescales in a consistent way. The models are fitted to discrete data from rain‐gauges using the generalised method of moments (GMM), a maximum likelihood method being impracticable. Such a model is used in the Weather Generator tool of the UK Climate Projections (UKCP09) project (Jones *et al.*, 2009), and examples of other applications are numerous (e.g. Khaliq & Cunnane (1996); Smithers *et al.* (2002); and Vandenberghe *et al.* (2011)).

However, a key limitation of existing point process‐based rainfall models is their assumed stationarity. The only nonstationary feature that these models can incorporate is seasonality, which is achieved by fitting separate models for each calendar month or season. In this paper, we improve upon existing methods by enabling covariate information to be incorporated, thereby allowing the models to be used for climate impact studies. The covariates used in practice within our method are simulated from advanced climate models, such as GCMs. We develop a new statistical downscaling method suitable for fine‐scale resolution by using a nonparametric kernel‐based approach to relate the parameters of a clustered point process‐based model to large‐scale atmospheric covariates, such as sea‐level pressure and temperature. These may be used because, unlike rainfall, the local variation in these variables is small, and their modelled values are considered reliable. Calendar month can still be included within the new framework, but may become redundant given the inclusion of the atmospheric variables, or be deliberately excluded if it is believed that seasonality in the future will be different from the past. The new methodology proposed combines a local mean or Nadaraya–Watson (Nadaraya, 1964; Watson, 1964) estimator with the GMM fitting approach. Extension to a local linear or higher dimension polynomial is possible and also considered.

The idea of local polynomial regression has been around for a long time, proposed originally by Cleveland (1979) and other authors, and there is a wealth of literature in the field [e.g. Fan & Gijbels (1996) and Wand & Jones (1995)]. Extensions to the original idea that have been useful in developing our methodology include local likelihood‐based methods (Tibshirani & Hastie, 1987) and local estimating equations (Carroll *et al.*, 1998). Our local mean method is effectively an application of the local mean GMM approach of Lewbel (2007), except that we do not assume that the bias term is zero in the asymptotic distribution of the parameter estimators. The local linear model requires a slightly different approach.

The new method is demonstrated here using a time series of 5‐min rainfall data from Bochum in Germany, a monthly sample of which is illustrated in Figure 1.

In order to focus on the fitting methodology, we consider the simplest version of the Bartlett‐Lewis clustered model at a single site. However, the methodology can readily be extended to the slightly more complex models in current use and potentially also to the spatial‐temporal domain.

## 2 Specification of the point process‐based rainfall model

In the basic Bartlett‐Lewis rectangular pulse (BLRP) model (Rodriguez‐Iturbe *et al.*, 1987), the point process is a clustered Poisson process. Rain‐events or ‘storms’ arrive in a Poisson process of rate *λ*, and each event generates a cluster of cell arrivals, with the time intervals between successive cells assumed to be independent, identically distributed random variables. It is normally assumed that the intervals between cells are exponentially distributed, so that the cell arrivals constitute a secondary Poisson process of rate *β*.

**Figure 2:**
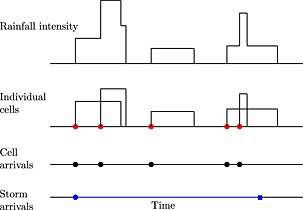
Illustration of a single storm of the Bartlett‐Lewis rectangular pulse model

Each cell has a random duration, during which it rains with a constant intensity, *X*, hence the description of the cells as ‘rectangular’. In the simplest version of the model, both the duration and the intensity are assumed to be exponentially distributed with parameters *η* and 1/*μ*_*X*_, respectively, and are independent of each other. The cell arrival process terminates after a time that is also exponentially distributed, with rate *γ*. This basic version thus has five parameters in total, (summarised in Table 1). Both storms and cells may overlap, and the total intensity of rain at any point in time is given by the sum of the intensities of all cells active at that time. The process in respect of a single storm is illustrated in Figure 2. Additional flexibility can be added by allowing a different distribution for cell intensities.

**Table 1:** Parameters of the Bartlett‐Lewis rectangular pulse model

Parameter	Definition
*λ*	Storm arrival rate
*β*	Cell arrival rate
*γ*	Storm termination rate
*η*	Cell termination rate
*μ*_*X*_	Mean cell intensity

## 3 Fitting methodology

### 3.1 Existing fitting approach, using GMM

GMM estimation requires a set of ‘population moment conditions’. For the rainfall models, these relate a vector of summary statistics of the time series of rainfall totals to the vector of their expected values under the model, with the dimension of the vector greater than or equal to the number of model parameters. The summary statistics are typically second‐order and third‐order sample moments at various temporal scales.

Because of seasonality, it is common to fit a separate model for each calendar month, treating the data from different years for a given calendar month as independent replicates. Assume we have a time‐series of, say 5‐minute, rainfall totals over a period of *n* months, with ***Y***_*t*_ defined as the vector of all the rainfall data in month *t*. We first calculate separate vectors of summary statistics, ***T*(*Y*_1_)**…***T*(*Y*_*n*_)**, for each of the *n* months of the data. The estimator, ${\hat{\theta }}_{m}$, in respect of calendar month, *m*, is then given by

(1) $$\begin{array}{cc} {\hat{\theta }}_{m} & ={\text{argmin}}_{\theta_{m}}\left[{\left\{\frac{1}{\overset{n}{\underset{t=1}{\sum }}I(m_{t}=m)}\overset{n}{\underset{t=1}{\sum }}I(m_{t}=m)\left[T(Y_{t})-\tau (\theta_{m})\right]\right\}}^{T}\right.\\ \;\;\;\left.\times W_{n}(m)\left\{\frac{1}{\overset{n}{\underset{t=1}{\sum }}I(m_{t}=m)}\overset{n}{\underset{t=1}{\sum }}I(m_{t}=m)[T(Y_{t})-\tau (\theta_{m})]\right\}\right] \end{array}$$

where argmin_***θ***_ means the value of ***θ*** that minimises the expression, *m*_*t*_ is the calendar month of the *t*th month, ***τ***(***θ***_*m*_) is the vector of expected values for calendar month *m* and ***W***_*n*_(*m*) is a positive‐definite weighting matrix for calendar month *m*, which ensures that we put a positive nonzero weight on all the moment conditions. *I* is the indicator function, such that *I*(*x*) = 1 if *x* is true, and 0 otherwise, so that we have $\overset{n}{\underset{t=1}{\sum }}I(m_{t}=m)$ months of data for calendar month *m*. This sum is just equal to *n*/12 if the data span a whole number of years, but in this form, the equation anticipates the extension to a more general covariate. The matrix, ***W***_*n*_(*m*), may depend on the data, but in order for the estimator to be well‐defined, it is required to converge in probability to a positive‐definite matrix of constants.

The estimator is consistent, provided certain regularity conditions are met (Hansen (1982); Hall (2005)). It can be shown that the optimal weights matrix, ***W***_*n*_(*m*) (in terms of minimising the variance of the parameter estimates), is the inverse of the covariance matrix of the fitting properties, which here must be estimated empirically due to the complexity of the analytical expressions. The approach of first calculating the fitting statistics separately for each month allows estimation of the required sample covariance matrix for each calendar month, as

(2) $$\mathrm{Var}[T(Y)|\;m]=\frac{\overset{n}{\underset{t=1}{\sum }}I(m_{t}=m)[T(Y_{t})-{\bar{T}}_{m}]{\left[T(Y_{t})-{\bar{T}}_{m}\right]}^{T}}{\overset{n}{\underset{t=1}{\sum }}I(m_{t}=m)-1}$$

where ${\bar{T}}_{m}$ is the mean of the statistics over calendar month *m*. Jesus & Chandler (2011) find that a two‐step approach is required in order to derive a reliable sample estimate of the full covariance matrix, but that the diagonal matrix of inverse variances (i.e. ignoring the correlations between the fitting statistics), calculated using just a single step, is close to optimal. The two‐step approach would involve simulating a large set of artificial data using an initial estimate of ***θ***_*m*_ (obtained using the simpler diagonal weighting matrix), then refitting the model using a full weighting matrix calculated from the simulated data. The drawback with this approach is the significant addition to computation time.

The choice of which and how many statistics to include is a fairly subjective one, although in practice rather restricted if it is deemed essential that analytical expressions be available for ***τ***(***θ***). Statistics are required at a range of aggregation levels, in order to reflect the observed structure of the rainfall process. In the empirical work of this paper described in Section 4, models are fitted using the mean hourly rainfall, plus the coefficient of variation, lag‐1 autocorrelation and skewness of rainfall totals at resolutions of 5 min and 1, 6 and 24 h (using analytical expressions derived by Rodriguez‐Iturbe *et al.* (1987) and Wheater *et al.* (2006)). Thus, ***T*** and ***τ*** are vectors of dimension 13.

Equation (1) is solved using numerical optimisation techniques, and the objective function is parameterized with the logarithms of the rainfall model parameters. This ensures that the fitted parameters are positive and has also been found to improve the stability of the numerical optimisation. Thus, for the five‐parameter BLRP model, the parameter vector is given by ***θ*** = (log*λ*, log*μ*_*X*_, log*β*, log*γ*, log*η*)^T^.

### 3.2 Local modelling

In this section, we show how the method may be adapted for one or more continuous covariates. Initially, and for the asymptotic derivations, we consider a single covariate, with extension to multiple covariates considered in Section 3.7. To motivate the development, we assume that this single covariate, denoted *X*_*t*_, is temperature, and that ***Y***_*t*_ is a time‐series of 5‐min rainfall totals over month *t*, as before. First, a suitable time interval needs to be determined over which to measure the covariates and calculate the corresponding rainfall statistics. It is already common practice to calculate statistics separately for each month (denoted by *t* in the equations), as discussed earlier, and this is a natural choice, which has many advantages. It is short enough that it is reasonable to treat the series within each interval as stationary. On the other hand, sample autocorrelations for monthly rainfall series tend to be very small, so a month is long enough to permit treatment of the data as independent between intervals. It is also long enough for any small sample biases in the statistics to be negligible, which is an issue primarily for statistics at the daily timescale. Additionally, mean monthly values of many atmospheric variables are readily available. Although detail of individual weather systems is clearly lost at a monthly timescale, this is not important as the aim is for the simulations to exhibit realistic rainfall behaviour within a gradually changing climate, rather than to reproduce actual historical rainfall patterns.

Assuming a single monthly continuous covariate (here temperature) at the evaluation point, *x*_0_, a natural extension of the existing method replaces the indicator functions of Equation (1) with kernel weights, which allow the parameter estimates to change smoothly with the value of the covariate. Thus, the estimator of the parameter vector at a given covariate value *X* = *x*_0_ is given by

(3) $$\begin{array}{cc} \hat{\theta }(x_{0}) & ={\text{argmin}}_{\theta_{x_{0}}}\left[\left\{\right.\frac{1}{\overset{n}{\underset{t=1}{\sum }}K_{h}(X_{t}-x_{0})}\overset{n}{\underset{t=1}{\sum }}K_{h}(X_{t}-x_{0})\;[T(Y_{t})-\tau (\theta (x_{0}))]{\}}^{T}\right.\\ \;\;\;\;\left.\times W_{n}(x_{0})\;\left\{\frac{1}{\overset{n}{\underset{t=1}{\sum }}K_{h}(X_{t}-x_{0})}\overset{n}{\underset{t=1}{\sum }}K_{h}(X_{t}-x_{0})\;[T(Y_{t})-\tau (\theta (x_{0}))]\right\}\right] \end{array}$$

where *K*_*h*_(*X*_*t*_−*x*_0_) = *h* ^−1^ *K*{(*X*_*t*_−*x*_0_)/*h*}, and *K*(·) is a kernel function, which is usually chosen to be a symmetric density function which integrates to 1, and is scaled to have a variance of 1. The expression has been written with the scaling factor $1/\overset{n}{\underset{t=1}{\sum }}K_{h}(X_{t}-x_{0})$ in order to highlight the similarity with Equation (1), but this is usually replaced by 1/*n* which does not affect the solution. The ‘tuning parameter’ or ‘bandwidth’, *h*, determines the size of the local neighbourhood and thus controls the amount of smoothing. The matrix ***W***_*n*_(*x*_0_) (discussed further in Section 3.4) depends on the covariate value *X* = *x*_0_, and converges to a positive‐definite matrix of constants, which we denote by ***W***(*x*_0_). The evaluation points can coincide with the observed covariate values, which permits an assessment of the goodness of fit. Alternatively, an arbitrary set of points can be used, for example the set of future values projected by a GCM or other climate model. In the latter case, though, care should be taken over any parts of the range that are sparsely represented in the observed data.

The choice of kernel function is relatively unimportant compared with the choice of *h* (Wand & Jones, 1995), and the Gaussian kernel function, given by $K(t)={\left(\sqrt{2\pi }\right)}^{-1}\exp(-t^{2}/2)$, is often used for convenience (and has been used in the empirical investigation in this paper), so that *K*_*h*_(*X*_*t*_−*x*_0_) is the normal density function with mean *x*_0_ and standard deviation *h*. The tuning parameter may alternatively be defined as a ‘span’, that is a fixed percentage of the data that contributes a positive weight to each local fit, in which case the kernel function must have compact support.

Kernel estimators are biased, with the bandwidth (or span) controlling the bias–variance trade‐off. Equation (3) assumes that the parameters are locally constant, but the approach may be extended to local linear or indeed to any order of polynomial. The complexity of the model is determined both by the bandwidth (the smaller the neighbourhood, the greater the effective number of parameters in the model) and by the order of the polynomial chosen. Ultimately, an appropriate compromise must be reached, and much of the kernel smoothing literature addresses the issues of selection of the optimal order and bandwidth. For the point process‐based rainfall models, although there are potential advantages to assuming a local linear approach, the additional complexities involved make order zero a sensible starting point. In the next section, we consider the asymptotic distribution of the local mean estimator, with the choice of weighting matrix and bandwidth discussed in Sections 3.4 and 3.5, respectively. Extension to local linear GMM is then considered in Section 3.6. The results of applying the proposed methodology will be discussed in Section 4.

### 3.3 Asymptotic distribution

In the Supporting information, we show that the local mean estimator, $\hat{\theta }(x_{0})$, is consistent for ***θ***_0_(*x*_0_), the true value of the parameter vector, and derive the asymptotic variance and bias. From these and appealing to a form of the central limit theorem (Schuster, 1972), it follows that the asymptotic distribution of $\hat{\theta }(x_{0})$ is given by

(4) $${\left(\mathrm{nh}\right)}^{1/2}\left\{\hat{\theta }(x_{0})-\theta_{0}(x_{0})-h^{2}B(x_{0})\right\}\to^{D}\;N(0,\mathrm{Var}[\hat{\theta }(x_{0})])$$

where the variance, $\mathrm{Var}[\hat{\theta }(x_{0})]$, at *x*_*o*_ is given by

(5) $$\begin{array}{cc} \mathrm{Var}[\hat{\theta }(x_{0})] & =\frac{1}{f(x_{0})}\;\int K^{2}(z)\;dz\;{\left\{{\left[\frac{\partial \tau (\theta_{0}(x_{0}))}{\partial \theta }\right]}^{T}\;W(x_{0})\frac{\partial \tau (\theta_{0}(x_{0}))}{\partial \theta }\right\}}^{-1}\;\;\;{\left[\frac{\partial \tau (\theta_{0}(x_{0}))}{\partial \theta }\right]}^{T}\;\;W(x_{0})\;\\ \;\times V(x_{0})\;W(x_{0})\frac{\partial \tau (\theta_{0}(x_{0}))}{\partial \theta }\;{\left\{{\left[\frac{\partial \tau (\theta_{0}(x_{0}))}{\partial \theta }\right]}^{T}\;W(x_{0})\frac{\partial \tau (\theta_{0}(x_{0}))}{\partial \theta }\right\}}^{-1} \end{array}$$

and the bias, *h* ^2^ ***B***(*x*_0_), by

(6) $$\begin{array}{cc} h^{2}\;B(x_{0}) & =h^{2}\int \;K(z)z^{2}\;dz\;\;{\left\{{\left[\frac{\partial \tau (\theta_{0}(x_{0}))}{\partial \theta }\right]}^{T}\;W(x_{0})\frac{\partial \tau (\theta_{0}(x_{0}))}{\partial \theta }\right\}}^{-1}\;{\left[\frac{\partial \tau (\theta_{0}(x_{0}))}{\partial \theta }\right]}^{T}\;W(x_{0})\;\\ \;\times \left\{\left[\right.\frac{1}{2}\;\frac{d}{dx}\left[\right.\frac{\partial \tau (\theta_{0}(x_{0}))}{\partial \theta }]+\frac{\partial \tau (\theta_{0}(x_{0}))}{\partial \theta }\frac{f^{\prime }(x_{0})}{f(x_{0})}]\;\theta_{0}^{\prime }(x_{0})+\frac{1}{2}\;\frac{\partial \tau (\theta_{0}(x_{0}))}{\partial \theta }\;\theta_{0}^{\prime \prime }(x_{0})\right\} \end{array}$$

where ***V***(*x*_0_) = Var[***T***(***Y***)|*X* = *x*_0_], and *f*(*x*) denotes the probability density function of the covariate *x*.

Although of interest in identifying the key drivers behind the behaviour of the local estimators and in informing the choice of bandwidth (discussed in Section 3.5), these asymptotic derivations are not directly useful in practical applications. The problem is that the expressions involve a number of unknown terms, including the ‘design density’, *f*, and its derivative and the first and second derivatives of ***θ*** with respect to *x*. Obtaining estimates of these is challenging even in the context of a simple local mean scatterplot smoother, and infeasible for the far more complex point process‐based models.

For the fit described subsequently, we adopt an alternative ‘quasi‐asymptotic’ approach, which involves replacing expressions in the asymptotic bias and variance with appropriate sample summations. This approach is advocated by both Fan & Gijbels (1995) (in the context of local polynomial regression) and Carroll *et al.* (1998) (in the context of local estimating equations), for making no more use of asymptotics than needed. Replacing ***θ***_0_(*x*_0_) with $\hat{\theta }(x_{0})$ and ***W***(*x*_0_) with ***W***_*n*_(*x*_0_) in the asymptotic variance, we get the ‘sandwich’ formula:

(7) $$\begin{array}{cc} \mathrm{Var}[\hat{\theta }(x_{0})] & \approx \frac{\overset{n}{\underset{t=1}{\sum }}K_{h}^{2}(X_{t}-x_{0})}{{\left\{\overset{n}{\underset{t=1}{\sum }}K_{h}(X_{t}-x_{0})\right\}}^{2}}\\ \;\times {\left[{\left[\frac{\partial \tau (\hat{\theta }(x_{0}))}{\partial \theta }\right]}^{T}\;W_{n}(x_{0})\frac{\partial \tau (\hat{\theta }(x_{0}))}{\partial \theta }\right]}^{-1}\;{\left[\frac{\partial \tau (\hat{\theta }(x_{0}))}{\partial \theta }\right]}^{T}\;W_{n}(x_{0})\;\mathrm{Var}[T(Y)|\;X=x_{0}]\\ \;\times W_{n}(x_{0})\;\frac{\partial \tau (\hat{\theta }(x_{0}))}{\partial \theta }\;{\left[{\left[\frac{\partial \tau (\hat{\theta }(x_{0}))}{\partial \theta }\right]}^{T}\;W_{n}(x_{0})\frac{\partial \tau (\hat{\theta }(x_{0}))}{\partial \theta }\right]}^{-1} \end{array}$$

The conditional variance of ***T***(***Y***) can be estimated at each required value of *X* using a local mean estimator with some fixed bandwidth h* as follows:

(8) $$\mathrm{Var}[T(Y)|\;X=x_{0}]\approx \frac{\overset{n}{\underset{t=1}{\sum }}K_{h^{*}}(X_{t}-x_{0})\left[T(Y_{t})-\tau (\overset{˘}{\theta }(x_{t}))\right]{\left[T(Y_{t})-\tau (\overset{˘}{\theta }(x_{t}))\right]}^{T}}{\overset{n}{\underset{t=1}{\sum }}K_{h^{*}}(X_{t}-x_{0})}$$

where $\overset{˘}{\theta }$ denotes the parameter vector fitted with the bandwidth h*.

A similar approach may be taken for the bias, to give

(9) $$\begin{array}{cc} \text{Bias}[\hat{\theta }(x_{0})] & \approx {\left[{\left[\frac{\partial \tau (\hat{\theta }(x_{0}))}{\partial \theta }\right]}^{T}\;W_{n}(x_{0})\frac{\partial \tau (\hat{\theta }(x_{0}))}{\partial \theta }\right]}^{-1}\;{\left[\frac{\partial \tau (\hat{\theta }(x_{0}))}{\partial \theta }\right]}^{T}\;W_{n}(x_{0})\\ \;\times \frac{\overset{n}{\underset{t=1}{\sum }}K_{h}(X_{t}-x_{0})\;\left[\tau (\hat{\theta }(x_{t}))-\tau (\hat{\theta }(x_{0}))\right]}{\overset{n}{\underset{t=1}{\sum }}K_{h}(X_{t}-x_{0})} \end{array}$$

This fairly crude approach to the calculation of bias is similar to that used in deriving the bias‐corrected‘twicing estimator’ of Stuetzle & Mittal (1979) for ordinary kernel regression and of Kauermann *et al.* (1998) for local estimating equations. Ruppert (1997) proposes a more sophisticated empirical approach based on the asymptotic theory, which involves approximating the bias by a polynomial function *f*(*h*,*γ*), the parameters of which are found using least‐squares estimation.

The ‘quasi‐asymptotic’ estimate of the variance, together with the asymptotic normality of the estimators, can be used to calculate approximate pointwise confidence intervals, although these will be for E$\left(\hat{\theta }(x_{0})\right)$ rather than ***θ***(*x*_0_) itself, due to the bias. In order to avoid confusion, such intervals are referred to as ‘variability bands’, rather than confidence intervals (Bowman & Azzalini, 1997). Although it is possible to estimate the bias term, as we have seen, this would itself involve terms in $\hat{\theta }$, and so would increase the variance of the estimator from that given in Equation (4).

### 3.4 The weighting matrix

So far, we have assumed only that the weighting matrix ***W***_*n*_(*x*_0_) may depend on the data, and that it converges to the positive‐definite nonrandom matrix, ***W***(*x*_0_). If, however, the weighting matrix is the inverse of the conditional covariance matrix of the statistics, that is ***W***(*x*_0_) = ***V***(*x*_0_)^−1^, then the expression for the variance of the asymptotic distribution of $\hat{\theta }(x_{0})$ simplifies, giving

(10) $$\begin{array}{c} {\left(\mathrm{nh}\right)}^{1/2}\left\{\hat{\theta }(x_{0})-\theta_{0}(x_{0})-h^{2}B(x_{0})\right\}\to^{D}\\ \;\;\;\;\;N\left(0,\;\frac{1}{f(x_{0})}\;\int K^{2}(z)\;dz\;{\left\{{\left[\frac{\partial \tau (\theta_{0}(x_{0}))}{\partial \theta }\right]}^{T}\;V{\left(x_{0}\right)}^{-1}\frac{\partial \tau (\theta_{0}(x_{0}))}{\partial \theta }\right\}}^{-1}\right) \end{array}$$

This choice for the weighting matrix gives optimal efficiency, and a two‐step procedure can be used, as described in Section 3.1, in respect of standard GMM. However, as discussed, the conclusions of Jesus & Chandler, 2011's 2011) simulation study justify using just a single step with the diagonal matrix of inverse variances. In order to reduce the computational burden (which is more onerous for local estimation), we have therefore chosen to use this simpler choice in estimating ***W***(*x*_0_) here. With a continuous covariate, there is only a single observation at each evaluation point, so a straightforward sample estimate cannot be used (as is possible with month as covariate). The practical approach used here involves grouping the data into a number of bins based on the value of the covariate, calculating sample variances within each bin (as in Equation ((2), but now conditioning on the covariate bin, rather than the calendar month) and treating these as the sample variances conditional on the mean covariate value of each of the bins. Variances conditional on other values of the covariate are then derived using a Nadaraya–Watson estimator. The fits were not found to be overly sensitive to the bandwidth used in this smoothing, which was thus selected subjectively.

### 3.5 Choosing a bandwidth

The issues involved in choosing a bandwidth for our model are the same as for local regression, although the greater complexity tends to suggest one of the simpler approaches. For the empirical study, for simplicity, we use a global bandwidth, which means that *h* is constant across the whole data range. A constant global bandwidth should be adequate for relatively smooth curves or where the amount of data is not sufficient to justify a local approach (see Fan & Gijbels (1995) for details of an approach to selecting a variable bandwidth).

How should a suitable global bandwidth be chosen? In the case of local regression, the choice is often subjective. More formally, automatic bandwidth selection methods generally aim to minimise the integrated mean squared error (IMSE) or a proxy for this, where the IMSE is given by $\text{IMSE}=\int [\text{Bias}{\left(\hat{\theta }(x)\right)}^{2}+\mathrm{Var}(\hat{\theta }(x))]\;f(x)dx$. The main options include the ‘plug‐in’ method, which involves the minimisation of the asymptotic expression for the IMSE, and some form of cross‐validation. We have chosen to use the latter as it is straightforward to apply and, unlike the ‘plug‐in’ method, does not require estimation of any additional parameters. The subject of bandwidth selection has been much discussed in the literature—see, for example (Loader, 1999) for more details and a comparison of these two methods.

Because of computational time constraints, repeated random subsampling is preferred to leave‐one‐out cross‐validation. This involves randomly splitting the data into test and training sets a number of times. For each such split, the model is fitted to the test data points using just the training observations and the bandwidth identified that gives the lowest error over the test data. The error here is defined as the mean weighted sum of squared residuals, that is as

(11) $$n_{\mathrm{ts}}^{-1}\overset{n_{\mathrm{ts}}}{\underset{t=1}{\sum }}\overset{k}{\underset{i=1}{\sum }}{\left[T_{i}(Y_{t})-\tau_{i}({\hat{\theta }}_{t_{h,\mathrm{tr}}})\right]}^{2}w_{t_{i}}$$

where *n*_*ts*_ is the number of observations in the test set, with $\hat{\theta }$ based on observations in the training set (denoted *tr*) and with the weights, $w_{t_{i}}$, based on the smoothed sample variances of the statistics as before. The optimal bandwidth is calculated for each such split—a histogram or density plot of the results across all the splits then also provides useful insight, which is another appealing feature of this method. The final choice of bandwidth is based on consideration of the mean or the median, in conjunction with subjective assessment.

### 3.6 Extension to local linear estimation

The Nadaraya–Watson or local mean approach suffers from some well‐known limitations. ‘Boundary bias’ arises at (or near) the boundaries where the observation points in the local neighbourhood lie only (or primarily) to one side, and the fitted curve tends to be too flat as a result. Another problem, ‘design bias’, arises when the design is not equi‐spaced. These types of bias are eliminated, at least asymptotically, by extending to a local linear approach, described by Fan (1992) as ‘design adaptive’. However, Ruppert & Wand (1994) caution against taking the local linear estimator as the automatic benchmark. While the two methods have identical asymptotic variances at interior points, at the boundaries and in finite samples, the Nadaraya–Watson estimator tends to have a smaller variance. Thus, in cases where the regression function is fairly flat, the Nadaraya–Watson estimator may have the advantage.

For the local mean estimator at the evaluation point *x*_0_, the approximation for ***θ***(*X*_*t*_) in the local neighbourhood of *x*_0_ is given by the constant ***θ***(*x*_0_). For the local linear case, the approximation is given instead by ***ψ***(*X*_*t*_) = ***θ***(*x*_0_) + ***θ***′(*x*_0_)(*X*_*t*_−*x*_0_). For ease of manipulation, we write the parameter set as a vector of length 2*q* given by ***b***(*x*) = (***θ***(*x*)^T^,***θ***′(*x*)^T^)^T^. The first set of *q* elements of $\hat{b}(x_{0})$ estimate the components of ***θ*** at *x*_0_, while the second set provide estimates of the gradients of the curves at *x*_0_.

In order to specify conditions for the gradient, as well as the value, of ***θ*** at *x*_0_, we apply the kernel weights to the quadratic form, rather than to the statistics, to give

(12) $$\hat{\theta }(x_{0})={\text{argmin}}_{\theta (x_{0})}\left\{\frac{1}{n}\overset{n}{\underset{t=1}{\sum }}\;K_{h}(X_{t}-x_{0})\;{\left[T(Y_{t})-\tau (\psi (X_{t}))\right]}^{T}\;W_{n}(x_{0})\;[T(Y_{t})-\tau (\psi (X_{t}))]\right\}$$

where ***ψ***(*X*_*t*_) = ***θ***(*x*_0_) + ***θ***′(*x*_0_)(*X*_*t*_−*x*_0_). Differentiating this equation with respect to ***b*** gives the following two sets of equations:

(13) $$0=\frac{1}{n}\overset{n}{\underset{t=1}{\sum }}K_{h}(X_{t}-x_{0})\;{\left[\frac{\partial \tau (\psi (X_{t}))}{\partial \theta }\right]}^{T}W_{n}(x_{0})\;[T(Y_{t})-\tau (\psi (X_{t}))]$$

(14) $$0=\frac{1}{n}\overset{n}{\underset{t=1}{\sum }}K_{h}(X_{t}-x_{0})\;(X_{t}-x_{0})\;{\left[\frac{\partial \tau (\psi (X_{t}))}{\partial \theta }\right]}^{T}W_{n}(x_{0})\;[T(Y_{t})-\tau (\psi (X_{t}))]$$

These equations exactly identify the 2*q* parameters and are effectively the sample equivalents of the required moment conditions.

Derivations of the asymptotic variance and bias follow a similar approach to the local mean case, using Taylor series expansions, and are not shown here. The asymptotic variance of $\hat{\theta }(x_{0})$ is found to be the same as for the local mean case, whereas the asymptotic bias is now given by

(15) $$\begin{array}{cc} \text{Bias}[\hat{\theta }(x_{0})] & \approx {\left\{{\left[\frac{\partial \tau (\theta_{0}(x_{0}))}{\partial \theta }\right]}^{T}W(x_{0})\frac{\partial \tau (\theta_{0}(x_{0}))}{\partial \theta }\right\}}^{-1}\;\\ \;\times \;\frac{1}{2}h^{2}\int K(z)z^{2}dz{\left[\frac{\partial \tau (\theta_{0}(x_{0}))}{\partial \theta }\right]}^{T}W(x_{0})\left[\;\frac{\partial \tau (\theta_{0}(x_{0}))}{\partial \theta }\;\;\theta_{0}^{\prime \prime }(x_{0})\right] \end{array}$$

It can be seen that the expression for the bias is much simpler, with the bias no longer dependent on the gradient of ***θ***, nor on the design density.

### 3.7 Multiple covariates

So far, a univariate covariate has been assumed. In principle at least, generalisation to multidimensional ***X*** is straightforward (see, e.g. Ruppert & Wand (1994) who consider multivariate local regression). The local mean model can be expressed as before, but now ***X*** is a *d*‐dimensional vector, that is $X_{t}={\left(X_{t_{1}},\ldots ,X_{t_{d}}\right)}^{T}$. In order to define neighbourhoods in *d* dimensions, a *d* × *d* symmetric positive‐definite smoothing matrix, ***H***, and a *d*‐dimensional kernel function, ***K***, are required. Wand and Jones (1995; Chapter 4) discuss various levels of sophistication when specifying the bandwidth matrix ***H***, which controls both the size and the direction of smoothing. A straightforward, but still flexible, approach is taken here with ***H*** constrained to be diagonal. This allows different degrees of smoothing to be applied to the different covariates in the directions of the coordinate axes. We can then take ***K*_*H*_**(***X***_*t*_−***x***_0_) as a ‘product kernel’ of the form

(16) $$K_{H}(X_{t}-x_{0})=K_{h_{1}}(X_{t_{1}}-x_{01})\;K_{h_{2}}(X_{t_{2}}-x_{02})...\;K_{h_{d}}(X_{t_{d}}-x_{0d})$$

To find an optimal bandwidth matrix, cross‐validation techniques can be used, as for a single covariate.

The main problem with multiple polynomial regression is what has been termed the ‘curse of dimensionality’ (Bellman, 1961), which simply means that as the dimension of the covariate vector increases for a fixed data set, the data become increasingly sparse in the sense that there are either very few points in the local neighbourhood or the neighbourhood ceases to be very ‘local’. The dimension therefore either needs to be kept appropriately low (typically limited in practice to two or three variables) or constraints need to be introduced in the model to reduce the effective dimensionality.

The asymptotic expressions for the variance and bias could in principle be derived. However, rather than using asymptotic expressions, we recommend estimating the variance using the sandwich method. No additional theory is then required, and the single Gaussian kernel in our equations is simply replaced by the product of individual kernels in respect of each required covariate, as in Equation (16). Extension to the local linear case is also possible.

## 4 Empirical Investigation

### 4.1 Motivation

In this section, we demonstrate the fitting methodology. The empirical investigation relates to a time series of 5‐min rainfall totals from Bochum in Germany, running over the 69 years from January 1931 to December 1999. In order for the methodology to be of practical use, it is important that any predictors selected will be well represented by climate models. The fitted rainfall model may then be conditioned on series of the covariates output under different greenhouse gas emission scenarios, allowing design implications of changing rainfall patterns to be assessed. For the modelling methodology to be successful, there must also exist reasonably strong relationships between at least some of our fitting properties and the predictors. An implicit assumption (common to all statistical downscaling techniques) is that these observed, empirical relationships remain valid under future climate conditions. The covariates we consider, in addition to calendar month, are monthly mean values from the National Centre for Environmental Prediction reanalysis data (Kalnay *et al.*, 1996; Kistler *et al.*, 2001) for the grid point with latitude 52.5′N and longitude 7.5′E. The reanalysis project uses an analysis/forecast system to perform data assimilation using historical data from 1948 to the present. This includes an extensive set of weather variables at a range of heights in the atmosphere on a 2.5°×2.5° grid covering the whole globe. These are available from January 1948, giving a total of 624 monthly observations (52 years) when combined with the time span of the Bochum rainfall series. Combinations of one, two or three covariates from the following were considered: sea‐level pressure, surface temperature, relative humidity at the surface, specific humidity at 700 hPa and the zonal (west–east) component of wind velocity. These were selected for more detailed investigation, after consideration of the statistical downscaling literature, the physical rainfall process, correlations between potential covariates and the fitting statistics and correlations between the potential covariates themselves. Given that seasonal behaviour is expected to change under the impact of climate change, we would prefer to capture seasonal behaviour implicitly through the atmospheric covariates. However, calendar month has also been included in the evaluation, using the von Mises distribution for smoothing instead of the Gaussian, to allow for its periodic nature.

### 4.2 Local mean v local linear, single covariate

**Figure 3:**
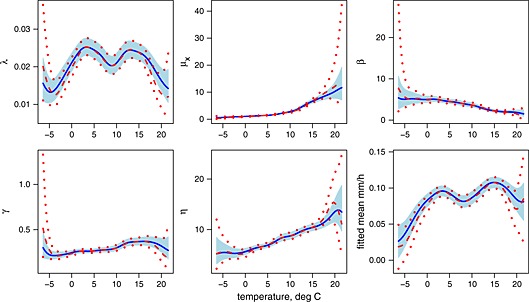
Fitted parameters with local mean (solid) and local linear (dashed) fits. The fitted mean is also shown. The covariate is temperature, with a bandwidth of 1.5° C. Variability bands plotted at ±2 standard errors (shaded for local mean and shown by dotted lines for local linear)

Initial analysis focused on the single covariate, temperature, in order to validate and refine the methodology and to decide between the local mean and local linear approaches. In order to keep run times relatively short, this initial comparison was carried out over an equally spaced grid of 60 temperature values, rather than over all 624 observed data points. Figure 3 shows the fitted parameters (plus the fitted mean hourly rainfall) for the two different orders of fit. Variability bands of ±2 standard errors, calculated using the sandwich method described in Section 3.3, are also shown. The standard errors for the fitted mean hourly rainfall have been calculated using the delta method approximation. The bandwidth at this stage has been chosen subjectively. The results are broadly in line with expectations, with the local linear and local mean fitted curves similar for interior points, but the local linear curve generally much steeper near the boundaries. Parameter uncertainty is relatively high close to the boundaries because of the low number of observation points, and particularly so for the local linear fit.

Computation times for the 60 evaluation points were of the order of an hour for the local linear estimator, compared with less than a minute for the local mean approach (both sets run on a laptop with a 2.10 GHz processor).

However, it is not obvious that the local linear fit is preferable here, even if the computation speed were not an issue. This is because the sparsity of observation points at very low and very high mean temperatures, coupled with the high variability of the statistics themselves, means that our confidence that the observed statistics at the boundaries are representative is fairly low. In the local linear approach, the effective sample sizes close to the boundaries are substantially lower, dramatically increasing the parameter uncertainty, as we have seen. In such cases, assuming a flatter curve may actually be preferable. Of course, this decision may depend on location and climate. However, it is also likely that computation time and difficulties with numerical optimisation will increase with the number of covariates, and/or with a more complex model. For these reasons, our preference here is for the local mean approach.

### 4.3 Choice of bandwidths

In order to choose optimal bandwidths, we initially considered each of the univariate predictors and used repeated random subsampling with 25 repetitions, as described in Section 3.5. For each repetition, the 624 observations were randomly split into 225 in the test data set and 399 in the training set. This split of 36%/64% is in line with the recommendations of Hengartner & Wegkamp (2002), who suggest taking the size of the testing sample as *n* ^*β*^ where *n* is the size of the training sample and *β* is in the range 0.8–0.95.

**Figure 4:**
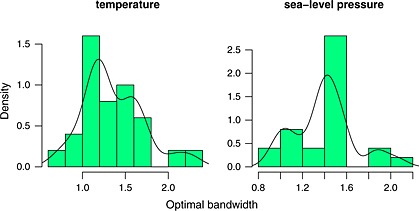
Density of the optimal bandwidth for each univariate predictor, based on 25 hold‐out samples, each of 225 observations

For each covariate, a grid of potential values of the bandwidth is required. Such grids typically have a geometric progression. Here, we first took a wide, but relatively coarse grid over a single sample to get an idea of the approximate location of the optimal bandwidth and the shape and steepness of the curve. We then carried out the 25 repetitions over a finer, but narrower grid. The finer grid had 27 points, with *h*_min_=10 × (*X*_(*n*)_−*X*_(1)_)/*n*, *h*_max_=(*X*_(*n*)_−*X*_(1)_)/5 and *h*_*j*_=1.1^*j*^ *h*_min_ for *j* = 1,…,25. Histograms of the optimal bandwidth based on the 25 hold‐out samples are given in Figure 4 for two of the potential predictors, temperature and sea‐level pressure.

Given the high level of noise in the observed data, it is to be expected that there will be a certain amount of variability in the ‘optimal’ bandwidth, derived over different subsets of the data and, as can be seen, this varies with the predictor. Nevertheless, the appropriate ballpark levels for all the predictors are reasonably clear. After inspection of the resulting curves, we select bandwidths of 1.3°C and 1.5 mb for temperature and sea‐level pressure, respectively.

For fits with multiple covariates, the optimal bandwidths are found in a similar way, but, in order to ensure manageable computation times, selection was restricted to a simple rescaling of the diagonal bandwidth matrix containing the optimal bandwidths for the appropriate univariate predictors. For a fit with the two covariates, temperature and sea‐level pressure, the bandwidths ultimately selected were 1.75°C and 2 mb, respectively.

### 4.4 Model selection and performance

In order to determine the optimal choice of covariates (and bearing in mind the requirement to keep the covariate dimension relatively low), our model selection approach broadly follows that used in the selection of the optimal bandwidth, that is we compare the mean weighted sum of squared errors of the 13 estimated statistics, over the 25 hold‐out samples, with the ‘optimal’ bandwidths used for each covariate set. Now, however, the weights, $w_{t_{i}}$, used in the calculation of the error statistic are based on the unconditional empirical variances of the statistics, because a fair comparison requires the same weights to be used for all covariate options. In order to assess the impact of the local fitting methodology compared with current practice, results for a global model (i.e. with no covariates) and with calendar month as a discrete covariate are also considered.

We are interested in the relative differences between the error statistics, rather than their actual values (and these cannot be taken as estimates of prediction error, as they are not based on an independent dataset). The model comparison indicates that using month as a covariate, as is common practice, reduces the median error statistic over the 25 samples by around 13% compared with just fitting a global model. Using an optimal bandwidth and the von Mises kernel, we found an improved reduction of 16%, just behind the optimal single covariate, temperature, which gave an improved reduction of 17% compared with no covariates. However, once temperature is already included in the model, month is found to add no further benefit, and the best second covariate is sea‐level pressure, giving a substantial further improvement: an overall reduction of 25% compared to the global model. Further improvement from the addition of a third covariate, is more limited, reducing the error by a further 2%. The optimal third choice, the zonal wind component, reflects its low correlation with the other covariates. Adding month as a third covariate was actually found to increase the error slightly. These are encouraging results. The ability to replace month with other covariates potentially has value even if the level of prediction error is broadly the same, because it allows climate change impacts to be modelled with different future seasonal behaviours. Here, we have shown that this approach can in fact also lead to a notable improvement in fit, allowing more realistic simulations to be generated from the model even if climate change is not a concern.

**Figure 5:**
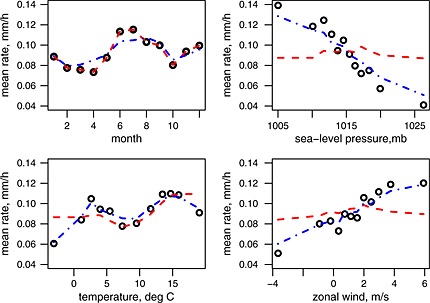
Mean rainfall intensity v binned values of selected covariates. Observed values are shown as circles; all the plots show values from two fitted Bartlett‐Lewis rectangular pulse models—one with covariate calendar month (dashed line) and the other with three covariates: sea‐level pressure, temperature and zonal wind (dot‐dashed line). In each case, the values of covariates not shown on the plot are at their observed values

In the hydrological literature, the fit of the point process‐based rainfall models is usually assessed by plotting mean values of various statistics of interest over each calendar month. Here, as well as considering the performance by month, we are also interested in how the observed and fitted properties vary over the atmospheric covariates. In order to produce comparable plots, both the observed and fitted statistics of interest are averaged over binned values of each of the continuous covariates in turn (using 12 bins with equal numbers in each bin). As an example, the plots in respect of the mean rainfall intensity are shown in Figure 5. All four plots in the figure show the two different models—one with the discrete covariate calendar month (dashed line) and the other with the three optimal covariates (dot‐dashed line). In each case, in order to permit comparison against the observations (o circles), the values of covariates not shown on the plot are at their observed values.

The current practice of a separate model for each calendar month reproduces the monthly means exactly. It also gives a reasonable fit across the temperature bands, except at the lowest and highest values. If, however, the seasonal pattern of temperatures in the future is different to that in the data, then simulations from this model will not correctly reflect this. The mean rainfall can be seen to show much greater variation over the sea‐level pressure bins than over calendar month or the temperature bins, and this variation is not reflected at all in the current approach. The model with the optimal three covariates: sea‐level pressure, temperature and zonal wind velocity reflects the variation in mean rainfall intensity well across the temperature, sea‐level pressure and wind velocity bands, in addition to capturing the broad seasonal effect. Plots of other fitting statistics are not included for the sake of brevity—the fit of the basic point process‐based models against various rainfall properties, including for example the moments, rainfall event profiles and extremes at varying resolutions have been well‐documented in the literature [e.g. Rodriguez‐Iturbe *et al.* (1988); Khaliq & Cunnane (1996); Smithers *et al.* (2002); Onof *et al.* (1996)]. All such properties will be more realistically modelled under potential climate change, with the inclusion of atmospheric covariates.

**Figure 6:**
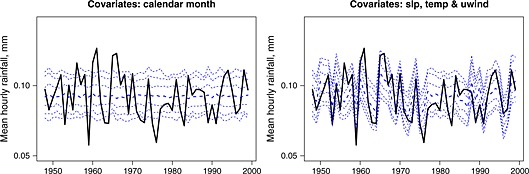
Simulated distributions of mean hourly rainfall (mm) for Bochum. The dashed bands show the 5th, 10th, 25th, 50th (thicker line), 75th, 90th and 95th percentiles from 200 simulations, and the solid line shows the observed values

Finally in this section, we consider interannual variability, the underestimation of which is one of the criticisms of many rainfall models. Figure 6 shows a range of percentiles between the 5th and the 95th in respect of the mean hourly rainfall, based on 200 simulations, over 52 years. In the first graph, the simulations have been based on the 12 monthly sets of parameters, sampling from the appropriate calendar month's parameter distribution for each observation month in turn. In the second graph, a different parameter distribution has been used for each observation month, reflecting that month's covariate values. It can be seen that allowing the parameters to depend on these covariates gives a much improved representation of the interannual variability.

### 4.5 Parameter estimates and uncertainty: higher dimensions

**Figure 7:**
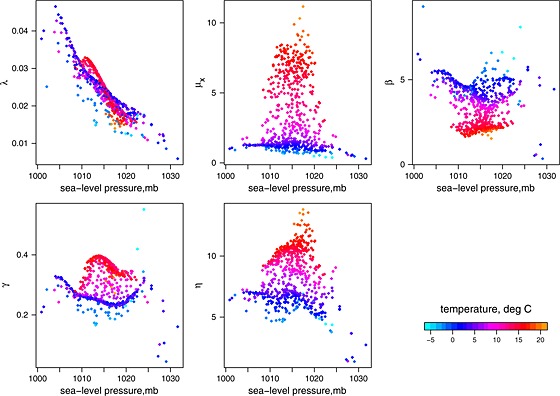
Scatterplots of fitted parameters versus sea‐level pressure and temperature. Bandwidths are sea‐level pressure 2.0 mb and temperature 1.75° C

Fitted parameters with the best combination of two covariates, sea‐level pressure and temperature are shown in Figure 7. Scatterplots are preferred here as a means of illustrating the relationships, as they also highlight where data are sparse, and do not show fits at unobserved covariate points. In order to make it easier to identify interesting relationships, the axes should also be exchanged, but here only a single set of graphs is shown.

Examining the results from the plotted fits in the context of physical weather processes, we recall that the covariates are monthly means, and so details of individual weather events are not captured. Rainfall intensity, *μ*_*X*_, can be seen to increase with temperature, which is in line with intuition, because increased temperatures lead to greater moisture content in the atmosphere and increased convective activity. Higher temperatures affect convective rainfall, not just because of increased moisture in the air, but also because the strength of updraughts is increased as the land is subject to greater heating. As temperatures increase, we also see that storms are generally shorter (*γ* tends to be higher). There are also fewer cells expected per rain event (given by 1 + *β*/*γ* in the BLRP model), and the cells are shorter. These effects occur at all levels of sea‐level pressure and tie in with the fact that convective storms tend to have fewer cells than the alternative lighter, long‐duration ‘stratiform’ rainfall. The storm arrival rate, *λ*, decreases almost linearly with increasing sea‐level pressure, at all temperatures, that is increased pressure tends to result in fewer storms. Again, this is in line with intuition, because rainfall is related to low‐pressure systems.

In higher dimensions, although pointwise variability bands are straightforward to calculate, it is harder to show the uncertainty in a way that is readily interpretable. One useful approach is to consider the ‘effective sample size’ in respect of each fit. Consider again the equation for the variance, given by Equation (7). In the case of the discrete covariate, calendar month, the sample size in respect of month *m* is clearly just *n*_*m*_. The equation for the variance in this case is given by

$$\begin{array}{cc} \mathrm{Var}[\hat{\theta }(x_{0})] & \approx \left[\right.\left[\right.\frac{\partial \tau ({\hat{\theta }}_{m})}{\partial \theta }{]}^{T}\;W_{n}(m)\frac{\partial \tau ({\hat{\theta }}_{m})}{\partial \theta }{]}^{-1}\;\left[\right.\frac{\partial \tau ({\hat{\theta }}_{m})}{\partial \theta }{]}^{T}\;W_{n}(m)\;\frac{\mathrm{Var}[T(Y)|\;m]}{n_{m}}\\ \;\times \;W_{n}(m)\;\frac{\partial \tau ({\hat{\theta }}_{m})}{\partial \theta }\;\left[\right.\left[\right.\frac{\partial \tau ({\hat{\theta }}_{m})}{\partial \theta }{]}^{T}\;W_{n}(m)\frac{\partial \tau ({\hat{\theta }}_{m})}{\partial \theta }{]}^{-1} \end{array}$$

By analogy, comparing with Equation (7), the inverse of the expression $\overset{n}{\underset{t=1}{\sum }}K_{h}^{2}(X_{t}-x_{0})/{\left\{\overset{n}{\underset{t=1}{\sum }}K_{h}(X_{t}-x_{0})\right\}}^{2}$ can be treated as the effective sample size for a continuous covariate at the observation with covariate value *x*_0_.

**Figure 8:**
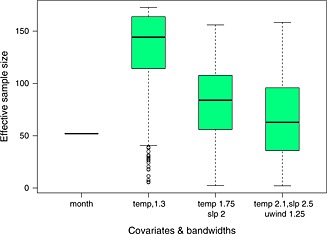
Effective sample sizes (in terms of number of observation months) for selected models and all modelled observation points. temp, temperature; slp, sea‐level pressure; uwind, zonal wind velocity

Figure 8 shows a boxplot of the results using this approach, across all observation points for each of the models. This shows that, at the (approximately) mean‐square optimal bandwidths, the effective sample sizes are generally reasonable for the majority of points and indeed higher than for the current approach of fitting by month. Clearly, some care needs to be taken with the fits at some observation points, particularly those with two or three dimensional covariates, but it should be clear which these are. Possibly, fitted models from other locations with slightly different ranges of the covariates could be used to supplement the sparse information here, or alternatively the bandwidth close to the boundaries could be increased (although of course this would lead to a corresponding increase in bias).

## 5 Discussion and Conclusions

In this paper, we have demonstrated how local smoothing ideas may be applied to relate the parameters of a complex multiparameter model to one or more continuous covariates. In particular, we have shown that a local GMM methodology offers a useful new approach to fitting point process‐based rainfall models. With just two or three covariates, we can produce a model with better explanatory power than the current approach, with more realistic interannual variation and with the ability to generate simulations that reflect future climate change scenarios. Our method could thus be used to explore the effects of climate change on rainfall and run‐off by simulating rainfall under a specific climate change scenario and comparing statistics of interest with a control period. We believe that this development will be useful to the hydrological community and to other practitioners who require artificial rainfall simulations for their applications.

We chose to use kernel smoothing, as local averaging is conceptually a natural extension of the current approach of averaging over a discrete covariate. The local mean method is simple and intuitive, may be implemented fairly quickly using existing software for fitting rainfall models and gives reasonable computation times. Further, the theoretical analysis and inference are straightforward, and the methodology allows estimation of uncertainty. We found that the local linear method, usually preferred for its superior performance at the boundaries, was not viable for our rainfall models, because of significantly increased computation speeds, coupled with sparse observations near the boundaries leading to extremely high parameter uncertainty.

An alternative approach to kernel smoothing would be to estimate *θ* as a linear combination of a collection (or ‘basis set’) of local functions of one or more covariates. Splines are typically chosen as the local functions. These are piecewise polynomials, with smoothness constraints at the joins or ‘knots’. An advantage of this approach is that it allows additive terms (without any need for backfitting), thus addressing the curse of dimensionality, which is a constraint for the local mean model, limiting the number of covariates that we can reasonably incorporate to a maximum of three. The idea of penalised splines is to use a large number of functions in the basis set to allow flexibility, but combine this with a roughness penalty to control the degree of smoothness. A choice of basis set and penalty is required. We have carried out some initial investigations using the approach of Eilers & Marx (1996). This uses equally spaced knots with a B‐spline (de Boor, 2001) basis set, which has desirable properties in terms of numerical stability. The roughness penalty is a multiple of the (usually second‐order) squared differences between coefficients. This multiple is effectively the smoothing parameter, playing a similar role to the bandwidth in the local mean method, although it is less easy to interpret intuitively.

Using this alternative spline‐based approach, much of the methodology described in the paper remains valid, but now the fitted values at the observed points are all found simultaneously, and the objective function includes a penalty term. The objective function must be minimised numerically, as before, and cross validation can be used to determine smoothing parameters. Our initial investigation has been promising, with results for one or two covariates similar to the local mean fits. Another potential advantage of the spline‐based approach is the ability to allow a different level of smoothing for different parameters, or indeed to fit some parameters parametrically. However, there is a cost to increased flexibility in terms of computational time, numerical stability and ease of implementation. Fitting times were found to be much slower than for the local mean method, although, unlike the local linear fits, not to such an extent as to make this a nonviable approach, particularly if the number of knots was not too large. A more detailed comparison between the two methods as well as consideration of uncertainty estimation for the spline‐based model would be an interesting topic for further research.

It should be noted, however, that our principal aim is for a modelling methodology that is useful to practitioners and straightforward to implement. Therefore, any additional complexity, particularly where it leads to significantly increased computation times, should be carefully considered against any additional benefit that it generates. In the current context, given the uncertainties associated with future climate projections and the strong correlations between climate variables, it is not clear that more than three covariates are required. If increased complexity adds only marginal improvements to the explanatory power of the model, then our current approach would be preferred.

Rainfall data are often collected at a network of rain gauges. For simplicity, we have demonstrated the use of continuous covariates in fitting models for rainfall at a single spatial location. However, it should be computationally feasible to apply it in a spatial‐temporal context, using simple models for spatial‐temporal rainfall such as those discussed in Wheater *et al.* (2000; Chapter 5) or Cowpertwait (2006).

## Supporting information

Supporting info item
