# Supplementary material for: Local generalised method of moments: an application to point process‐based rainfall models
Source: Environmetrics. 2015 Mar 22;26(4):312–25. doi: 10.1002/env.2338 (PMC4975607; doi:10.1002/env.2338)
Supplement: Supplementary file 1 — Supporting info item [file ENV-26-312-s001.pdf]

Supplement to:

Local Generalised Method of Moments: An application to point process-based rainfall models

Jo Kaczmarska, Valerie Isham, Paul Northrop
Department of Statistical Science
University College London, Gower Street, London WC1E 6BT, UK.

S1 Introduction

This supplement provides the asymptotic derivations for the paper 'Local Generalised Method of Moments: An application to point process-based rainfall models'. We demonstrate the consistency of the local mean GMM estimator of the parameter vector of the point process-based rainfall model, and derive the asymptotic variance and bias. For ease of reference, we start by briefly restating the notation.

We assume we have a time-series of, say 5-minute, rainfall totals over a period of n months, with $\boldsymbol{Y}_t$ defined as the vector of all the rainfall data in month t. $\boldsymbol{T(Y_1)\dots T(Y_n)}$ represent vectors of summary statistics for each of the n months of the data. The parameter vector, denoted $\boldsymbol{\theta}$, is assumed to be a function of a single covariate, X. The vector of expected values of the statistics at the covariate value x under the model is denoted $\boldsymbol{\tau}(\boldsymbol{\theta}(x))$. $K()$ is a kernel function, with $K_h(X_t - x) = h^{-1}K\{(X_t - x)/h\}$, where h is the bandwidth.

S2 Asymptotic derivations: consistency, variance and bias

Throughout the derivations, we assume that the regularity conditions required for standard GMM hold (see, for example, Jesus and Chandler (2011)), except that now we condition on the covariate, X. Conditions on $\boldsymbol{\tau}$ are that it is twice differentiable with respect to $\boldsymbol{\theta}$, the parameter vector, and that the derivatives are bounded, for all $\boldsymbol{\theta}$ such that $\boldsymbol{\theta}(x) \in \boldsymbol{\Theta(x)}$, a compact subset of $\mathbb{R}^q$. It is also required that $\partial\boldsymbol{\tau}/\partial\boldsymbol{\theta}$ be of full rank.

In addition, various smoothness conditions are required in order to allow local averaging. Here we assume that the following functions are sufficiently smooth in a neighbourhood of x_0 to permit differentiation as required, and that the functions and derivatives are finite at x_0: the parameter vector function, $\boldsymbol{\theta}(x)$, the design density, $f(x)$, the conditional variance $\text{Var}[\boldsymbol{T}(\boldsymbol{Y})|X = x]$, and the composite function $\boldsymbol{\tau}(\boldsymbol{\theta}(x))$. The last of these requires similar smoothness for $\boldsymbol{\tau}$ in $\boldsymbol{\theta}$. We assume also that $f(x_0) > 0$.

The kernel function is assumed to be a continuous, symmetric density function with $\int z^2 K(z)\,\mathrm{d}z = k_2 \neq 0$ and $\int z^{2r} K(z)\,\mathrm{d}z < \infty, \;\; r = 1, 2$. The kernel function does not need to be compactly supported, but should decay fast enough to eliminate the impact of a remote data point (Fan and Gijbels, 1996). Additional assumptions in order to simplify the asymptotic derivations are that X is scalar, and that the evaluation point, x_0, does not lie near the boundary of the design region.

We assume that there exists a unique, true value of the parameter vector, $\boldsymbol{\theta}_0(x) \in \boldsymbol{\Theta}(x)$, such that $\mathrm{E}[\boldsymbol{T}(\boldsymbol{Y})|X = x] = \boldsymbol{\tau}(\boldsymbol{\theta}_0(x))$ and define:

$$\boldsymbol{G}_n(\boldsymbol{\theta}(x)) = \frac{1}{n}\sum_{t=1}^{n} K_h(X_t - x)\,[\boldsymbol{T}(\boldsymbol{Y}_t) - \boldsymbol{\tau}(\boldsymbol{\theta}(x))], \tag{S1}$$

where t is the observation month, and the pairs $(X_t, \boldsymbol{Y}_t)$ are assumed to be independent and identically distributed as $(X, \boldsymbol{Y})$. The bandwidth is assumed to be a function of the sample size, n, although for notational simplicity we write h rather than h_n. Then the local GMM estimator at $x = x_0$ is given by:

$$\hat{\boldsymbol{\theta}}(x_0) = \mathrm{argmin}_{\{\boldsymbol{\theta}(x_0)\}}\, \boldsymbol{G}_n(\boldsymbol{\theta}(x_0))^{\mathrm{T}}\, \boldsymbol{W}_n(x_0)\, \boldsymbol{G}_n(\boldsymbol{\theta}(x_0)), \tag{S2}$$

where $\boldsymbol{W}_n(x_0)$ is a $k \times k$ positive-definite weighting matrix, with $\boldsymbol{W}_n(x_0) \to^p \boldsymbol{W}(x_0)$. For convenience, the sum of weights divisor of Equation (3) in the main paper is replaced by n here, which does not affect the solution. At $\boldsymbol{\theta}(x_0) = \hat{\boldsymbol{\theta}}(x_0)$ the derivative of the minimand in Equation (S2) is equal to zero. In this form, the equation is an example of an estimating equation, and is given by:

$$\begin{aligned} 0 &= \left[\frac{\partial \boldsymbol{G}_n(\hat{\boldsymbol{\theta}}(x_0))}{\partial \boldsymbol{\theta}}\right]^{\mathrm{T}} \boldsymbol{W}_n(x_0)\; G_n(\hat{\boldsymbol{\theta}}(x_0)) \qquad \text{(S3)}\\ &= \left\{\frac{1}{n}\sum_{t=1}^{n} K_h(X_t - x_0)\left[\frac{\partial \boldsymbol{\tau}(\hat{\boldsymbol{\theta}}(x_0))}{\partial \boldsymbol{\theta}}\right]\right\}^{\mathrm{T}} \boldsymbol{W}_n(x_0)\left\{\frac{1}{n}\sum_{t=1}^{n} K_h(X_t - x_0)[\boldsymbol{T}(\boldsymbol{Y}_t) - \boldsymbol{\tau}(\hat{\boldsymbol{\theta}}(x_0))]\right\}, \end{aligned}$$

where the notation $\partial \boldsymbol{G}_n(\hat{\boldsymbol{\theta}}(x_0))/\partial \boldsymbol{\theta}$ and $\partial \boldsymbol{\tau}(\hat{\boldsymbol{\theta}}(x_0))/\partial \boldsymbol{\theta}$ is used to represent $\partial \boldsymbol{G}_n/\partial \boldsymbol{\theta}|_{\boldsymbol{\theta}=\hat{\boldsymbol{\theta}}(x_0)}$ and $\partial \boldsymbol{\tau}/\partial \boldsymbol{\theta}|_{\boldsymbol{\theta}=\hat{\boldsymbol{\theta}}(x_0)}$ respectively (i.e. the Jacobian matrices of $\boldsymbol{G}_n$ and $\boldsymbol{\tau}$, evaluated at $\boldsymbol{\theta} = \hat{\boldsymbol{\theta}}(x_0)$).

In order for there to exist a unique value to which the estimator converges as the sample size increases, we require that $\boldsymbol{G}_n(\boldsymbol{\theta}(x_0))^{\mathrm{T}}\, \boldsymbol{W}_n(x_0)\, \boldsymbol{G}_n(\boldsymbol{\theta}(x_0))$ converges uniformly in probability to a non-random function which has a unique minimum at the true value $\boldsymbol{\theta}(x_0) = \boldsymbol{\theta}_0(x_0)$.

Consider first the asymptotic behaviour of $\boldsymbol{G}_n(\boldsymbol{\theta}(x_0))$. We have:

$$\begin{aligned} \mathrm{E}[\boldsymbol{G}_n(\boldsymbol{\theta}(x_0))] &= \mathrm{E}\left\{K_h(X - x_0)\;[\boldsymbol{T}(\boldsymbol{Y}) - \boldsymbol{\tau}(\boldsymbol{\theta}(x_0))]\right\}\\ &= \int \frac{1}{h} K\left(\frac{x - x_0}{h}\right) \mathrm{E}[\boldsymbol{T}(\boldsymbol{Y}) - \boldsymbol{\tau}(\boldsymbol{\theta}(x_0))|X = x]\; f(x)\; \mathrm{d}x. \qquad \text{(S4)} \end{aligned}$$

Letting $\boldsymbol{R}_{\boldsymbol{\theta}}(x) = \mathrm{E}[\boldsymbol{T}(\boldsymbol{Y}) - \boldsymbol{\tau}(\boldsymbol{\theta}(x_0))|X = x]$ and making the substitution $z = (x - x_0)/h$:

$$\begin{aligned} \mathrm{E}[\boldsymbol{G}_n(\boldsymbol{\theta}(x_0))] &= \int\; K(z)\; \boldsymbol{R}_{\boldsymbol{\theta}}(x_0 + zh)\; f(x_0 + zh)\mathrm{d}z\\ &= \boldsymbol{R}_{\boldsymbol{\theta}}(x_0) f(x_0) \;+\; h^2 \int\; K(z) z^2\, \mathrm{d}z\, \left\{\frac{1}{2}\boldsymbol{R}''_{\boldsymbol{\theta}}(x_0) f(x_0) + \boldsymbol{R}'_{\boldsymbol{\theta}}(x_0) f'(x_0)\right.\\ &\quad \left. + \frac{1}{2}\boldsymbol{R}_{\boldsymbol{\theta}}(x_0) f''(x_0)\right\} + o(h^2), \qquad \text{(S5)} \end{aligned}$$

where we have taken a Taylor series expansion of the product $\boldsymbol{R}_{\boldsymbol{\theta}}(x_0 + zh)\; f(x_0 + zh)$ about x_0, and noted that $\int K(z)\,\mathrm{d}z = 1$ and $\int K(z) z\; \mathrm{d}z = 0$. The expectation converges converges to $\boldsymbol{R}_{\boldsymbol{\theta}}(x_0) f(x_0)$ provided $h \to 0$.

Taking a similar approach for the variance of $\boldsymbol{G}_n(\boldsymbol{\theta}(x_0))$:

$$\begin{aligned}
&\mathrm{Var}[\boldsymbol{G}_n(\boldsymbol{\theta}(x_0))] \\
&= \frac{1}{n}\mathrm{Var}\left\{K_h(X-x_0)\ [\boldsymbol{T}(\boldsymbol{Y})-\boldsymbol{\tau}(\boldsymbol{\theta}(x_0))]\right\} \\
&= \frac{1}{n}\mathrm{E}\big\{K_h^2(X-x_0)[\boldsymbol{T}(\boldsymbol{Y})-\boldsymbol{\tau}(\boldsymbol{\theta}(x_0))]\ [\boldsymbol{T}(\boldsymbol{Y})-\boldsymbol{\tau}(\boldsymbol{\theta}(x_0))]^{\mathrm{T}}\big\} \\
&- \frac{1}{n}\mathrm{E}\big\{K_h(X-x_0)[\boldsymbol{T}(\boldsymbol{Y})-\boldsymbol{\tau}(\boldsymbol{\theta}(x_0))]\big\}\ \mathrm{E}\big\{K_h(X-x_0)[\boldsymbol{T}(\boldsymbol{Y})-\boldsymbol{\tau}(\boldsymbol{\theta}(x_0))]\big\}^{\mathrm{T}} \\
&= \frac{1}{n}\int\int K_h^2(x-x_0)\ [\boldsymbol{T}(\boldsymbol{y})-\boldsymbol{\tau}(\boldsymbol{\theta}(x_0))]\ [\boldsymbol{T}(\boldsymbol{y})-\boldsymbol{\tau}(\boldsymbol{\theta}(x_0))]^{\mathrm{T}}\ f(\boldsymbol{y}|x)\,f(x)\,\mathrm{d}\boldsymbol{y}\mathrm{d}x + O\Big(\frac{1}{n}\Big) \\
&= \frac{1}{n}\int \frac{1}{h^2}K^2\left(\frac{x-x_0}{h}\right)\ \Big[\mathrm{Var}[\boldsymbol{T}(\boldsymbol{Y})|X=x]+\boldsymbol{R}_{\boldsymbol{\theta}}(x)\boldsymbol{R}_{\boldsymbol{\theta}}(x)^{\mathrm{T}}\Big]\ f(x)\,\mathrm{d}x + O\Big(\frac{1}{n}\Big) \\
&= \frac{f(x_0)}{nh}\int K^2(z)\,\mathrm{d}z\ \Big[\mathrm{Var}[\boldsymbol{T}(\boldsymbol{Y})|X=x_0]+\boldsymbol{R}_{\boldsymbol{\theta}}(x_0)\boldsymbol{R}_{\boldsymbol{\theta}}(x_0)^{\mathrm{T}}\Big]\ + O\Big(\frac{1}{n}\Big), \qquad \text{(S6)}
\end{aligned}$$

where the last line follows using a zeroth order approximation of $\Big[\mathrm{Var}[\boldsymbol{T}(\boldsymbol{Y})|X=x_0+zh]+\boldsymbol{R}_{\boldsymbol{\theta}}(x_0+zh)\boldsymbol{R}_{\boldsymbol{\theta}}(x_0+zh)^{\mathrm{T}}\Big]\ f(x_0+zh)$ about x_0.

The variance converges to zero provided that $nh\to\infty$ as $n\to\infty$. Therefore, by the weak law of large numbers, $\boldsymbol{G}_n(\boldsymbol{\theta}(x_0))$ is a consistent estimator of $\boldsymbol{R}_{\boldsymbol{\theta}}(x_0)f(x_0)$ if $h\to 0$ and $nh\to\infty$.

Since $\boldsymbol{W}_n(x_0)\to^p \boldsymbol{W}(x_0)$, then, by Slutsky's theorem, $\boldsymbol{G}_n(\boldsymbol{\theta}(x_0))^{\mathrm{T}}\,\boldsymbol{W}_n(x_0)\,\boldsymbol{G}_n(\boldsymbol{\theta}(x_0))$ tends to a non-random function of $\boldsymbol{\theta}$, given by:

$$f^2(x_0)\boldsymbol{R}_{\boldsymbol{\theta}}(x_0)^{\mathrm{T}}\ \boldsymbol{W}(x_0)\boldsymbol{R}_{\boldsymbol{\theta}}(x_0). \qquad \text{(S7)}$$

We have shown pointwise convergence and assumed compactness of $\boldsymbol{\Theta}$. Stochastic equicontinuity is then a sufficient condition for the convergence to be uniform (Newey, 1991). Now $\partial\boldsymbol{G}_n(\boldsymbol{\theta}(x_0))/\partial\boldsymbol{\theta}$ is $O_p(1)$ and converges to $-\partial\boldsymbol{\tau}(\boldsymbol{\theta}_0(x_0))/\partial\boldsymbol{\theta}\,f(x_0)$, since $\partial\boldsymbol{\tau}(\boldsymbol{\theta}(x_0))/\partial\boldsymbol{\theta}$ is a finite matrix of constants and $n^{-1}\sum_{t=1}^{n}K_h(X_t-x_0)$ is just the standard density estimator of $f(x_0)$.

Then, by the Mean Value Theorem for vector valued functions of several variables (see, for example, Apelian and Surace (2009)):

$$\begin{aligned}
\boldsymbol{G}_n(\tilde{\boldsymbol{\theta}}(x_0))-\boldsymbol{G}_n(\boldsymbol{\theta}(x_0)) &= \begin{bmatrix} \frac{\partial \boldsymbol{G}_{n_1}(\boldsymbol{\vartheta}_1(x_0))}{\partial\boldsymbol{\theta}} \\ \vdots \\ \frac{\partial \boldsymbol{G}_{n_k}(\boldsymbol{\vartheta}_k(x_0))}{\partial\boldsymbol{\theta}} \end{bmatrix}\ [\tilde{\boldsymbol{\theta}}(x_0)-\boldsymbol{\theta}(x_0)] \qquad \text{(S8)} \\
&= \boldsymbol{D}_G(\boldsymbol{\vartheta}_1,\boldsymbol{\vartheta}_2,\ldots\boldsymbol{\vartheta}_k)\ [\tilde{\boldsymbol{\theta}}(x_0)-\boldsymbol{\theta}(x_0)],
\end{aligned}$$

say, where $\partial\boldsymbol{G}_{n_i}(\boldsymbol{\vartheta}_i(x_0))/\partial\boldsymbol{\theta}$ represents the ith row of the matrix $\partial\boldsymbol{G}_n/\partial\boldsymbol{\theta}$ evaluated at the point $\boldsymbol{\vartheta}_i$, which lies on the segment $(\tilde{\boldsymbol{\theta}},\boldsymbol{\theta})$ (which is assumed to be entirely contained within $\boldsymbol{\Theta}$). So:

$$\begin{aligned}
||G_n(\tilde{\boldsymbol{\theta}}(x_0))-G_n(\boldsymbol{\theta}(x_0))|| &= \Big|\Big|\boldsymbol{D}_G(\boldsymbol{\vartheta}_1,\boldsymbol{\vartheta}_2,\ldots\boldsymbol{\vartheta}_k)[\tilde{\boldsymbol{\theta}}(x_0)-\boldsymbol{\theta}(x_0)]\Big|\Big| \\
&\le ||\boldsymbol{D}_G(\boldsymbol{\vartheta}_1,\boldsymbol{\vartheta}_2,\ldots\boldsymbol{\vartheta}_k)||\ ||\tilde{\boldsymbol{\theta}}(x_0)-\boldsymbol{\theta}(x_0)|| \\
&\le \boldsymbol{M}\ ||\tilde{\boldsymbol{\theta}}(x_0)-\boldsymbol{\theta}(x_0)||, \qquad \text{(S9)}
\end{aligned}$$

where $\boldsymbol{M} = \max_{\boldsymbol{\vartheta}_1 \ldots \boldsymbol{\vartheta}_k \in \Theta} ||\boldsymbol{D}_G(\boldsymbol{\vartheta}_1, \boldsymbol{\vartheta}_2, \ldots \boldsymbol{\vartheta}_k)||$ is $O_p(1)$, as demonstrated earlier, and $||\boldsymbol{A}|| = \sqrt{\sum_{i,j} |a_{ij}|^2}$ (i.e. $||\cdot||$ represents the Euclidean matrix norm). This Lipschitz condition is sufficient for stochastic equicontinuity (Newey, 1991), and therefore implies uniform convergence, as required.

The limiting function (S7) (which only takes values greater than or equal to zero) has a unique minimum at the true value $\boldsymbol{\theta} = \boldsymbol{\theta}_0$, since $\boldsymbol{R}_{\boldsymbol{\theta}_0}(x_0) = 0$, and $\boldsymbol{R}_{\boldsymbol{\theta}}(x_0) \neq 0$ for $\boldsymbol{\theta} \neq \boldsymbol{\theta}_0$ (by the initial moment condition). These conditions mean that as $n \to \infty$ (with the additional proviso that $h \to 0$ and $nh \to \infty$), our estimating equation defines a unique estimator $\hat{\boldsymbol{\theta}}$ that is consistent for $\boldsymbol{\theta}$.

S2.1 Asymptotic Variance

Now we consider the asymptotic variance of the estimator. We apply the mean value theorem again, now for the line segment $(\hat{\boldsymbol{\theta}}, \boldsymbol{\theta}_0)$, so we have:

$$\begin{aligned} \boldsymbol{G}_n(\hat{\boldsymbol{\theta}}(x_0)) &= \boldsymbol{G}_n(\boldsymbol{\theta}_0(x_0)) + \begin{bmatrix} \frac{\partial \boldsymbol{G}_{n_1}(\check{\boldsymbol{\theta}}_1(x_0))}{\partial \boldsymbol{\theta}} \\ \vdots \\ \frac{\partial \boldsymbol{G}_{n_k}(\check{\boldsymbol{\theta}}_k(x_0))}{\partial \boldsymbol{\theta}} \end{bmatrix} [\hat{\boldsymbol{\theta}}(x_0) - \boldsymbol{\theta}_0(x_0)] \qquad \text{(S10)} \\ &= \boldsymbol{G}_n(\boldsymbol{\theta}_0(x_0)) + \boldsymbol{D}_G(\check{\boldsymbol{\theta}}_1, \check{\boldsymbol{\theta}}_2, \ldots \check{\boldsymbol{\theta}}_k)\ [\hat{\boldsymbol{\theta}}(x_0) - \boldsymbol{\theta}_0(x_0)], \end{aligned}$$

where the points $\check{\boldsymbol{\theta}}_1, \check{\boldsymbol{\theta}}_2, \ldots \check{\boldsymbol{\theta}}_k$ lie on the line segment $(\hat{\boldsymbol{\theta}}, \boldsymbol{\theta}_0)$. Substituting this into Equation (S3) gives:

$$0 = \left[\frac{\partial \boldsymbol{G}_n(\hat{\boldsymbol{\theta}}(x_0))}{\partial \boldsymbol{\theta}}\right]^{\mathrm{T}} \boldsymbol{W}_n(x_0)\ \left[\boldsymbol{G}_n(\boldsymbol{\theta}_0(x_0)) + \boldsymbol{D}_G(\check{\boldsymbol{\theta}}_1, \check{\boldsymbol{\theta}}_2, \ldots \check{\boldsymbol{\theta}}_k)\ [\hat{\boldsymbol{\theta}}(x_0) - \boldsymbol{\theta}_0(x_0)]\right]. \qquad \text{(S11)}$$

Since $\hat{\boldsymbol{\theta}}$ is consistent and converges to $\boldsymbol{\theta}_0$, then so do $\check{\boldsymbol{\theta}}_1, \check{\boldsymbol{\theta}}_2, \ldots \check{\boldsymbol{\theta}}_k$, as they lie on the segment $(\hat{\boldsymbol{\theta}}, \boldsymbol{\theta}_0)$. We have also shown that $\partial \boldsymbol{G}_n(\boldsymbol{\theta}(x_0))/\partial \boldsymbol{\theta}$ converges in probability to the non-random function $-\partial \boldsymbol{\tau}(\boldsymbol{\theta}_0(x_0))/\partial \boldsymbol{\theta}\, f(x_0)$, and we have $\boldsymbol{W}_n(x_0) \to^p \boldsymbol{W}(x_0)$ (by appropriate selection) and so this may be restated as:

$$-f(x_0)\left[\frac{\partial \boldsymbol{\tau}(\boldsymbol{\theta}_0(x_0))}{\partial \boldsymbol{\theta}}\right]^{\mathrm{T}} \boldsymbol{W}(x_0)\ \left[\boldsymbol{G}_n(\boldsymbol{\theta}_0(x_0)) - f(x_0)\left[\frac{\partial \boldsymbol{\tau}(\boldsymbol{\theta}_0(x_0))}{\partial \boldsymbol{\theta}}\right] [\hat{\boldsymbol{\theta}}(x_0) - \boldsymbol{\theta}_0(x_0)]\right] = o_p(1). \qquad \text{(S12)}$$

Since $\partial \boldsymbol{\tau}(\boldsymbol{\theta}_0(x_0))/\partial \boldsymbol{\theta}$ is of full rank, and $\boldsymbol{W}(x_0)$ is positive-definite, then $[\partial \boldsymbol{\tau}(\boldsymbol{\theta}_0(x_0))/\partial \boldsymbol{\theta}]^{\mathrm{T}}\, \boldsymbol{W}(x_0)$ $[\partial \boldsymbol{\tau}(\boldsymbol{\theta}_0(x_0))/\partial \boldsymbol{\theta}]$ is invertible, and we have:

$$\begin{aligned} \hat{\boldsymbol{\theta}}(x_0) - \boldsymbol{\theta}_0(x_0) &\approx \frac{1}{f(x_0)}\left\{\left[\frac{\partial \boldsymbol{\tau}(\boldsymbol{\theta}_0(x_0))}{\partial \boldsymbol{\theta}}\right]^{\mathrm{T}} \boldsymbol{W}(x_0)\frac{\partial \boldsymbol{\tau}(\boldsymbol{\theta}_0(x_0))}{\partial \boldsymbol{\theta}}\right\}^{-1} \\ &\quad \times \left[\frac{\partial \boldsymbol{\tau}(\boldsymbol{\theta}_0(x_0))}{\partial \boldsymbol{\theta}}\right]^{\mathrm{T}} \boldsymbol{W}(x_0)\boldsymbol{G}_n(\boldsymbol{\theta}_0(x_0)). \end{aligned} \qquad \text{(S13)}$$

From Equation (S6) above, noting that $\boldsymbol{R}_{\boldsymbol{\theta}_0}(x_0) = 0$ by the moment condition:

$$\mathrm{Var}[\boldsymbol{G}_n(\boldsymbol{\theta}_0(x_0))] \approx \frac{f(x_0)}{n\,h}\mathrm{Var}[\boldsymbol{T}(\boldsymbol{Y})|X = x_0] \int K^2(z)\mathrm{d}z.$$

So, returning to Equation (S13), the asymptotic expression for the variance of the estimator is given by:

$$\begin{aligned}\mathrm{Var}[\hat{\boldsymbol{\theta}}(x_0)] \approx{}& \frac{1}{n\,h f(x_0)} \int K^2(z)\,\mathrm{d}z \\ &\times \left\{ \left[\frac{\partial \boldsymbol{\tau}(\boldsymbol{\theta}_0(x_0))}{\partial \boldsymbol{\theta}}\right]^{\mathrm{T}} \boldsymbol{W}(x_0) \frac{\partial \boldsymbol{\tau}(\boldsymbol{\theta}_0(x_0))}{\partial \boldsymbol{\theta}} \right\}^{-1} \left[\frac{\partial \boldsymbol{\tau}(\boldsymbol{\theta}_0(x_0))}{\partial \boldsymbol{\theta}}\right]^{\mathrm{T}} \boldsymbol{W}(x_0) \\ &\times \mathrm{Var}[\boldsymbol{T}(\boldsymbol{Y})|X=x_0]\,\boldsymbol{W}(x_0) \frac{\partial \boldsymbol{\tau}(\boldsymbol{\theta}_0(x_0))}{\partial \boldsymbol{\theta}} \left\{ \left[\frac{\partial \boldsymbol{\tau}(\boldsymbol{\theta}_0(x_0))}{\partial \boldsymbol{\theta}}\right]^{\mathrm{T}} \boldsymbol{W}(x_0) \frac{\partial \boldsymbol{\tau}(\boldsymbol{\theta}_0(x_0))}{\partial \boldsymbol{\theta}} \right\}^{-1}.\end{aligned} \tag{S14}$$

S2.2 Asymptotic bias

Next we consider the bias, taking the expectation of both sides of Equation (S13) to get:

$$\begin{aligned}\mathrm{Bias}[\hat{\boldsymbol{\theta}}(x_0)] \approx{}& \frac{1}{f(x_0)} \left\{ \left[\frac{\partial \boldsymbol{\tau}(\boldsymbol{\theta}_0(x_0))}{\partial \boldsymbol{\theta}}\right]^{\mathrm{T}} \boldsymbol{W}(x_0) \frac{\partial \boldsymbol{\tau}(\boldsymbol{\theta}_0(x_0))}{\partial \boldsymbol{\theta}} \right\}^{-1} \\ &\times \left[\frac{\partial \boldsymbol{\tau}(\boldsymbol{\theta}_0(x_0))}{\partial \boldsymbol{\theta}}\right]^{\mathrm{T}} \boldsymbol{W}(x_0)\, \mathrm{E}[\boldsymbol{G}_n(\boldsymbol{\theta}_0(x_0))].\end{aligned} \tag{S15}$$

From Equation (S5), now putting $\boldsymbol{R}_{\boldsymbol{\theta}_0}(x_0) = 0$:

$$\mathrm{E}[\boldsymbol{G}_n(\boldsymbol{\theta}_0(x_0))] \approx h^2 \int K(z) z^2\,\mathrm{d}z \left\{ \frac{1}{2} \boldsymbol{R}''_{\boldsymbol{\theta}_0}(x_0) f(x_0) + \boldsymbol{R}'_{\boldsymbol{\theta}_0}(x_0) f'(x_0) \right\}. \tag{S16}$$

We have:

$$\begin{aligned}\boldsymbol{R}_{\boldsymbol{\theta}_0}(x) &= \mathrm{E}[\boldsymbol{T}(\boldsymbol{Y}) - \boldsymbol{\tau}(\boldsymbol{\theta}_0(x_0))\,|\,X=x] \\ &= \boldsymbol{\tau}(\boldsymbol{\theta}_0(x)) - \boldsymbol{\tau}(\boldsymbol{\theta}_0(x_0)).\end{aligned} \tag{S17}$$

Also:

$$\boldsymbol{R}'_{\boldsymbol{\theta}_0}(x) = \frac{\partial \boldsymbol{\tau}(\boldsymbol{\theta}_0(x))}{\partial \boldsymbol{\theta}}\ \boldsymbol{\theta}'_0(x) \tag{S18}$$

and

$$\boldsymbol{R}''_{\boldsymbol{\theta}_0}(x) = \frac{\mathrm{d}}{\mathrm{d}x}\left[\frac{\partial \boldsymbol{\tau}(\boldsymbol{\theta}_0(x))}{\partial \boldsymbol{\theta}}\right] \boldsymbol{\theta}'_0(x) + \frac{\partial \boldsymbol{\tau}(\boldsymbol{\theta}_0(x))}{\partial \boldsymbol{\theta}}\ \boldsymbol{\theta}''_0(x). \tag{S19}$$

Substituting back:

$$\begin{aligned}\mathrm{E}[\boldsymbol{G}_n(\boldsymbol{\theta}_0(x_0))] \approx{}& h^2 \int K(z) z^2\,\mathrm{d}z \\ &\times \left\{ \left[\frac{1}{2} \frac{\mathrm{d}}{\mathrm{d}x}\left[\frac{\partial \boldsymbol{\tau}(\boldsymbol{\theta}_0(x_0))}{\partial \boldsymbol{\theta}}\right] f(x_0) + \frac{\partial \boldsymbol{\tau}(\boldsymbol{\theta}_0(x_0))}{\partial \boldsymbol{\theta}} f'(x_0) \right] \boldsymbol{\theta}'_0(x_0) \right. \\ &\left. + \frac{1}{2} \frac{\partial \boldsymbol{\tau}(\boldsymbol{\theta}_0(x_0))}{\partial \boldsymbol{\theta}} f(x_0)\ \boldsymbol{\theta}''_0(x_0) \right\}.\end{aligned} \tag{S20}$$

We leave the first term in this form for ease of notation, since the second differential of $\boldsymbol{\tau}(\boldsymbol{\theta})$ with respect to $\boldsymbol{\theta}$ would give a three-dimensional array and consequent notational complexity. So finally, the asymptotic expression for the bias is given by:

$$\mathrm{Bias}[\hat{\boldsymbol{\theta}}(x_0)] \approx h^2 \int K(z) z^2 \, \mathrm{d}z \; \left\{ \left[\frac{\partial \boldsymbol{\tau}(\boldsymbol{\theta}_0(x_0))}{\partial \boldsymbol{\theta}} \right]^{\mathrm{T}} \boldsymbol{W}(x_0) \frac{\partial \boldsymbol{\tau}(\boldsymbol{\theta}_0(x_0))}{\partial \boldsymbol{\theta}} \right\}^{-1} \left[\frac{\partial \boldsymbol{\tau}(\boldsymbol{\theta}_0(x_0))}{\partial \boldsymbol{\theta}} \right]^{\mathrm{T}} \boldsymbol{W}(x_0) \times \left\{ \left[\frac{1}{2} \frac{\mathrm{d}}{\mathrm{d}x} \left[\frac{\partial \boldsymbol{\tau}(\boldsymbol{\theta}_0(x_0))}{\partial \boldsymbol{\theta}} \right] + \frac{\partial \boldsymbol{\tau}(\boldsymbol{\theta}_0(x_0))}{\partial \boldsymbol{\theta}} \frac{f'(x_0)}{f(x_0)} \right] \boldsymbol{\theta}_0'(x_0) + \frac{1}{2} \frac{\partial \boldsymbol{\tau}(\boldsymbol{\theta}_0(x_0))}{\partial \boldsymbol{\theta}} \boldsymbol{\theta}_0''(x_0) \right\}. \tag{S21}$$

References

Apelian, C. and Surace, S. (2009). *Real and Complex Analysis*. Pure and Applied Mathematics. Taylor & Francis.

Fan, J. and Gijbels, I. (1996). *Local Polynomial Modelling and its Applications*. Chapman and Hall, London.

Jesus, J. and Chandler, R. E. (2011). Estimating functions and the generalized method of moments. *Interface Focus*, 1(6):871–885. doi 10.1098/rsfs.2011.0057.

Newey, W. K. (1991). Uniform convergence in probability and stochastic equicontinuity. *Econometrica*, 59(4):1161–1167.
